# Unraveling R-loops: The hidden drivers of inflammation and immune dysregulation

**DOI:** 10.1097/MD.0000000000042833

**Published:** 2025-06-13

**Authors:** Fan Zhang, Dingyun Wang, Guodong Zhao, Dingmao Wang

**Affiliations:** a Department of Gastrointestinal Surgery, Haikou Affiliated Hospital of Central South University Xiangya School of Medcine, Haikou, China; b Department of Hepatobiliary Surgery, Haikou Affiliated Hospital of Central South University Xiangya School of Medcine, Haikou, China; c Department of Traditional Chinese Medicine, Haikou Affiliated Hospital of Central South University Xiangya School of Medcine, Haikou, China.

**Keywords:** antitumor immunity, congenital immunity, inflammatory responses, R-loop

## Abstract

An R-loop is a complex nucleic acid structure consisting of an RNA–DNA hybrid and an associated single-stranded DNA. This structure plays an important role in many biological processes, including gene regulation, DNA replication, and maintenance of genome stability. Under physiological conditions, the R-loop is in a dynamic balance of generation and decomposition, which is a complex process involving multiple mechanisms of its formation, clearance, and regulation. Emerging evidence indicates that R-loops can drive inflammation and participate in immune processes. This review summarizes immune dysregulation resulting from impaired R-loop clearance and analyzes recent advances in computational methods for R-loop regulation, aiming to identify new avenues for developing R-loop-targeted immunotherapies.

## 1. Introduction

An R-loop is a dynamic 3-stranded nucleic acid structure that is formed when an RNA strand invades the double-stranded DNA during transcription. It is composed of an RNA–DNA hybrid and a non-template double-stranded DNA at the corresponding position.^[1]^ R-loops were initially considered rare byproducts of transcription; however, continuous in-depth research has shown that they are widely present in the genomes of bacteria,^[2]^ yeast,^[3]^ and higher eukaryotes.^[4]^

In normal cells, R-loops are formed at the promoter and termination sites to regulate transcription and participate in various biological processes, leading to both positive and negative effects. Physiologically, R-loops regulate gene expression and promote DNA repair, transcription, and RNA processing.^[5,6]^ In addition, they can use the sequence specificity of RNA–DNA base pairing to exclude cofactors or target them to different chromosomal sites. A classic example from prokaryotes is the clusters of regularly interspaced short palindromic repeats-Cas9 system.^[7]^ However, when R-loop homeostasis is disturbed, the presence of R-loops can interfere with transcription initiation, elongation, or termination, leading to replication stress and genomic instability.^[8]^ Moreover, an abnormal number of R-loops and the subsequent activation of innate immunity may lead to many diseases, such as neurodegenerative diseases and cancer.^[9]^

R-loops have been shown to play a crucial role in immunity. Studies have reported that the occurrence of Aicardi–Goutières syndrome (AGS), a rare inflammatory disease, is closely related to R-loops.^[1]^ Excessive R-loops can induce necrosis of epithelial cells and lead to spontaneous intestinal inflammation.^[10]^ R-loops can regulate the recombination switch that occurs in the variant surface glycoprotein locus during immune evasion in trypanosomes.^[11]^ In addition, they can regulate antitumor immunity by affecting metabolic reprogramming,^[12]^ altering immune cell function,^[13]^ and regulating interferons,^[14]^ thereby influencing tumor progression.

Given the potential of R-loops to regulate cellular immunity, this review aimed to summarize the maintenance of R-loop homeostasis and the consequences of homeostatic imbalance and discuss the mechanisms through which R-loops contribute to inflammatory responses and antitumor immunity. Furthermore, this review discusses computational approaches that provide valuable insights into the genome-wide formation, distribution, and regulation of R-loops. We analyze advancements in these methods, focusing on modeling the mechanisms of R-loop formation and resolution, identifying regulatory factors, and constructing interaction networks. These computational insights not only pave the way for novel R-loop-targeted immunotherapies but also facilitate the screening and potential design of R-loop modulators/inhibitors.

Furthermore, this review discusses the distribution of R-loops in the genome, covering localization techniques, comparison of distribution in different organisms, and computational prediction methods. Subsequently, it deeply analyzes the progress in computational methods for R-loop regulation, such as the regulatory mechanisms of formation and resolution, identification of regulatory factors, and construction of network models. These findings not only provide new avenues for developing R-loop-targeted immunotherapies but also make the screening of R-loop inhibitors possible.

## 2. Maintenance of R-loop homeostasis

As a 3-stranded structure consisting of an RNA–DNA hybrid and a single-stranded DNA, the R-loop is ubiquitous in organisms from bacteria to mammals. The formation of an R-loop usually occurs during transcription, and its homeostasis is essential for cells to perform their normal functions. Therefore, disruption of R-loop homeostasis can lead to serious consequences. Under physiological conditions, R-loops are in a dynamic balance of generation and decomposition. In mammalian cells, approximately 300 R-loops are in a stable state.^[15]^ The clearance and stability of R-loops are regulated through various mechanisms involving various enzymes and proteins.

### 2.1. Scavenging effects of ribonucleases

Cells contain various ribonucleases that can specifically degrade RNA molecules. Through this degradation, the cells can eliminate unnecessary R-loops and maintain R-loop homeostasis. Ribonuclease H enzymes (RNase H1 and RNase H2) can recognize RNA–DNA hybrids and degrade the RNA involved in R-loops, thereby promoting the disassembly of the R-loops.^[16]^

When an R-loop is present, RNase H1 is recruited to the RNA–DNA hybrid through direct interaction with replication protein A, a single-stranded DNA-binding protein that covers displaced DNA strands in R-loops.^[17]^ TonEBP recruits METTL3 to R-loops through the Rel homology domain for m6A modification of R-loop RNA,^[18]^ whereas ARID1A recruits METTL3 and METTL14 to R-loops, leading to m6A modification of R-loop RNA^[19]^, which contributes to the recruitment of RNase H1 to R-loops. Subsequently, RNase H1 eliminates R-loops,^[16]^ by hydrolyzing the phosphodiester backbone of the RNA strand in the RNA–DNA hybrid.

Although RNase H2 shares some similarities with RNase H1, it is more efficient in cleaving RNA than RNaseH1. In particular, it can cleave RNA–DNA hybrids at a single ribonucleotide, successfully separating the RNA and DNA strands.^[20]^ RNase H1 and H2 are regulated through different mechanisms. RNase H2 relies on the cell cycle because it is essential for both R-loop processing and ribonucleotide excision repair in the G2/M phase. On the contrary, RNase H1 functions independently of the cell cycle to remove R-loops and appears to be activated under a high R-loop load.^[21]^

Recent studies have shown that Dicer can cleave the RNA strand in an R-loop.^[22]^ This finding suggests that RNases other than RNase H are also involved in R-loop processing.

### 2.2. DNA repair mechanisms

In addition to RNase H, DNA repair mechanisms play a crucial role in the elimination of R-loops and R-loop-induced damage by recognizing DNA damage caused by R-loop formation and initiating R-loop clearance.

Homologous recombination is one of the major mechanisms for repairing DNA double-strand breaks (DSBs). It can recognize and repair DSBs caused by accumulation of R-loops. As a key participant in homologous recombination, BRCA1 is not only involved in the repair of DSBs^[23]^ but also interacts with RNA polymerase II to promote R-loop clearance.^[24]^ Notably, accumulation of R-loops is more pronounced in BRCA2-deficient cells.^[25]^ Moreover, BRCA2 can interact with RNA polymerase II to regulate R-loops.^[26]^

Although the main role of nucleotide excision repair is to repair DNA damage caused by exposure to ultraviolet rays or chemicals, it also plays a role in the clearance of R-loops. The core components of NER, XPG, and XPF endonucleases, have been shown to participate in R-loop clearance. These enzymes can recognize structural abnormalities in DNA caused by R-loop formation and contribute to R-loop clearance by cleaving the DNA at the damaged site.^[27,28]^ Processing of R-loops by XPG and XPF is affected by the splicing factor XAB2^[29]^; however, the exact underlying mechanism remains unknown, suggesting a knowledge gap in this field.

## 3. Imbalance of R-loop homeostasis and inflammatory responses

The temporal and/or spatial accumulation of undesired R-loops in the genome exerts deleterious effects, such as transcription defects, transcription–replication conflicts, cell cycle arrest, and genomic instability. Genomic instability caused by R-loop imbalance can lead to abnormal immune responses, which may, in turn, trigger the development of autoimmune diseases.

### 3.1. R-loops are abnormally accumulated during inflammatory responses

The abnormal accumulation of R-loops has been reported in many studies on autoimmune diseases. AGS is a severe inflammatory disease in children that can lead to nervous system damage. The occurrence of AGS is closely related to autoimmunity.^[30]^ In a study, a genome-wide analysis of fibroblasts from patients with AGS showed an overall increase in R-loop levels. In addition, we have previously found that the types of R-loops formed in the cells of patients with AGS are different from naturally occurring R-loops.^[31]^ Moreover, diseases such as Sjogren syndrome and systemic lupus erythematosus are associated with abnormal R-loop formation, and patients with these diseases usually have high levels of R-loops and related inflammatory markers.^[32]^

Skin tissue contains a complex network of immune cells that are essential for host defense and tissue homeostasis.^[33]^ Exposure to intense UV light can lead to skin inflammation, which is often accompanied by an overall increase in R-loop levels.^[34]^ A study on patients with inflammatory bowel disease showed that R-loop levels were significantly high in inflamed tissues and this increase was caused by dysfunction of TDP-43.^[10]^ Furthermore, a study on artificial blood cells showed that R-loop accumulation and inflammatory signaling were upregulated in cells with reduced DDX41 expression^[35]^ These findings suggest that R-loop imbalance plays an important role in the pathogenesis of inflammation-related diseases and is involved in triggering typical immune response stimuli. Consequently, further research on R-loops and their influence on inflammatory responses is warranted.

### 3.2. Accumulation of R-loops can activate the inflammatory response

Both the mutations of R-loop regulators and their excessive accumulation can lead to inflammation. Therefore, understanding the mechanisms through which R-loops affect the inflammatory response is necessary. The cGAMP synthase (cGAS)–stimulator of interferon genes (STING) pathway, which is activated,^[36]^ when cGAS binds to dsDNA, is considered an important pathway in the production of inflammatory responses. Therefore, when R-loops accumulate, their byproducts can act as immunogenic substances to activate the downstream inflammatory signaling pathways. A study on artificial blood cells showed that excessive R-loops in DDX41 mutants triggered the cGAS–STING inflammatory pathway, leading to excessive proliferation of hematopoietic endothelial cells and hematopoietic stem and progenitor cells, which may contribute to the development of hematologic diseases.^[37]^ In a study on myelodysplastic syndrome, McLemore et al demonstrated that unresolved R-loops accumulated in the nucleus and cytoplasm of cell models of myelodysplastic syndrome and that cytoplasmic R-loops triggered immune responses through cGAS.^[38]^ The ring-splitting proteins senataxin and BRCA1 play an important role in preventing excessive accumulation of R-loops, and their deletion can directly lead to an increase in the number of R-loops in the nucleus and the formation of RNA–DNA hybrids. Upon entry into the cytoplasm, these hybrids trigger downstream IRF3 signaling via direct sensing of cGAS and TLR3, inducing an inflammatory response.^[9]^ These findings indicate that dsDNA in R-loops can activate the cGAS–STING pathway, leading to the upregulation of downstream Nf-κb and interferon genes, stimulation of the release of inflammatory factors, and production of an inflammatory response.

In addition to direct activation, R-loops can interfere with the normal process of transcription and affect the transcriptional activity of genes, leading to the overexpression of certain inflammation-related genes. This change in gene expression may contribute to the development of a chronic inflammatory state, which further affects the immune response. For example, after UV stimulation, a large number of pathological R-loops are generated in the nucleus of skin cells. These R-loops are considered redundant and are involved in HSF4–COIL complex-mediated regulation of transcription, which results in altered expression of target genes (ATG7, TFPI, and LIMS1) and contributes to inflammatory responses.^[34]^ Therefore, pathological accumulation of R-loops is a key step in the production of UV-induced inflammatory responses.

Notably, accumulation of R-loops is also associated with the impairment of autophagy. Autophagy is an important mechanism^[39]^ through which cells remove damaged and abnormal proteins. Excessive R-loops may inhibit autophagy, aggravating cellular damage, which in turn triggers an inflammatory response.^[40]^ R-loop accumulation and impaired autophagy have been shown to trigger cGAS-dependent inflammation in senataxin-deficient cells.^[35]^ The ATG7 gene encodes an E1-like activating enzyme that initiates classical autophagy by promoting the formation of autophagosomes.^[41]^ Autophagy can regulate the type I interferon response and inflammasome output, which are essential components of an inflammatory response.^[42]^ Therefore, the absence or dysfunction of autophagy may lead to immune cell hyperactivity, which in turn triggers chronic inflammation.^[43]^ Given that accumulation of R-loops may enhance ATG7 expression.^[34]^ R-loops may regulate autophagy and subsequently affect inflammatory responses by altering ATG7 expression.

Altogether, R-loops trigger inflammatory responses through various mechanisms, including perception of cellular stress, activation of immune responses, stimulation of cytokine release, and regulation of gene expression and autophagy. Understanding these mechanisms is important for the development of therapeutic strategies against chronic inflammatory and autoimmune diseases.

## 4. Imbalance of R-loop homeostasis and antitumor immunity

R-loops can accumulate in many types of cancer cells, such as prostate, colorectal, liver, and lung cancer cells. They can interfere with DNA repair pathways and activate oncogenes, leading to dysregulation of cell proliferation, genomic instability, and cancer development. However, the effects of R-loops on the tumor immune microenvironment remain unclear. In the following sections, we discuss the mechanisms through which R-loops affect antitumor immunity to create a favorable environment for the growth of cancer cells.

### 4.1. Disruption of R-loop homeostasis affects the tumor microenvironment

The tumor microenvironment (TME) plays a crucial role in the occurrence, development, and treatment of cancer. TME is a complex environment composed of tumor cells, immune cells, stromal cells, extracellular matrix, and other components. Its function and characteristics have a profound impact^[44]^ on the biological behavior of tumors. In recent years, the role of R-loops in the tumor immune microenvironment has attracted increased attention. The formation of R-loops may facilitate the escape of tumor cells from immune surveillance by inducing an immunosuppressive microenvironment. In a recent study, an R-loop score model was constructed using single-cell RNA-sequencing datasets of lung adenocarcinoma. The results showed that malignant cells with low R-loop scores exhibited activation of pathways related to glycolysis, epithelial–mesenchymal transition, and immune escape, which reduced therapeutic efficacy.^[12]^ Cell communication analysis showed that low R-loop scores led to a significant increase in depletion scores for CD4^+^ and CD8^+^ T cells and NK cells. In addition, the R-loop score model could effectively predict the response of patients to targeted therapy, chemotherapy, or immunotherapy in 32 independent cohorts, suggesting that the R-loop can be used as an independent factor to predict the immune status of patients with cancer. Furthermore, a study on desmoplastic small round cell tumors showed that R-loops affected PD-L1 expression on tumor cell surface through ataxia telangiectasia and Rad3-related, thereby attenuating the immune response to tumor cells.^[45]^

R-loops may affect immune evasion of tumor cells by altering cell–cell signaling. Cancer cells secrete various signaling molecules to communicate with surrounding cells, and the formation of an R-loop may alter the expression of these signaling molecules, affecting the activity and function of immune cells.^[46]^ In terms of antibody production by immune cells, maintenance of R-loop homeostasis plays an important role in the antibody diversity of B cells^[47]^ R-loops are involved in the production of antibodies in B cells and regulate the diversity of these antibodies. Disruption of R-loop homeostasis may reduce the efficiency of antibody production, which may attenuate the immune response.^[48]^

Therefore, individualized treatment strategies should be developed according to the levels of R-loops in the cells of the TME of patients with cancer. An evaluation of R-loop levels may help predict the response of patients to specific immunotherapies and optimize treatment strategies.

### 4.2. Application of R-loops in cancer immunotherapy

At present, R-loops are detected primarily using immunoprecipitation combined with deep sequencing and single-stranded DNA/RNA immunoprecipitation sequencing. These techniques can help assess the distribution of R-loops in the entire genome.^[49–51]^ Furthermore, the cleavage under targets and tagmentation (CUT and Tag) method can generate highly specific R-loop signals through specific digestion and tagging of R-loops, which is suitable for the detection of transient R-loops. Compared with traditional detection methods, the CUT&Tag method yields higher signal intensity, especially in the gene promoter region.^[52,53]^ Given that immunoprecipitation combined with deep sequencing, single-stranded DNA/RNA immunoprecipitation sequencing, and CUT and Tag can efficiently and accurately locate and quantify R-loops in cells, these techniques represent important tools for investigating the role of R-loops in tumors. With these techniques, the levels of R-loops in different tumor cells can be analyzed and the relationship between R-loops and immunotherapy response can be assessed. Malignant tumor cells can construct an immunosuppressive microenvironment that is conducive to cell growth by regulating R-loop formation. This microenvironment supports tumor proliferation and promotes the development of resistance to chemotherapy and immunotherapy.^[54]^ Therefore, targeting R-loops is an efficient strategy for inhibiting tumor cell growth and maximizing treatment efficacy. Although some studies have provided a basis for developing R-loop-based therapeutic strategies,^[55]^ further investigation is warranted to optimize these targeted strategies for clinical application.

## 5. Advancements in computational methods for R-loops

Studying the distribution of R-loops across different organisms helps reveal universal principles and species-specific characteristics of R-loop biology.^[56]^ Research indicates that repetitive elements associated with R-loop formation differ among various species. These repetitive elements might exert species-specific or developmental-stage-specific regulatory roles in R-loop formation, providing important clues for an in-depth understanding of R-loop function and evolution in diverse organisms. Furthermore, the association between R-loops and repetitive elements also varies in organisms at different developmental stages. Therefore, identifying a precise computational method is particularly crucial for studying the role of R-loops in immunity.

### 5.1. The rise of computational biology and the development of R-loop models

To investigate the formation mechanism(s) of genomic R-loops in depth, researchers have developed various computational models. To identify R-loops, Wongsurawat et al developed a computational algorithm and mapped R-loop forming sequences (RLFS) onto 66,803 sequences defined by UCSC as “known” genes. They found that approximately 59% of these transcribed sequences contained at least 1 RLFS. They created R-loopDB, a database that collects all identified RLFS from over half of human genes and links to the UCSC Genome Browser to facilitate information integration and visualization across various bioinformatics sources.^[57]^ Furthermore, Kuznetsov et al developed a quantitative model of R-loop-forming sequences, enabling genome-scale prediction of R-loops.^[58]^ Using this model, they predicted approximately 660,000 RLFS in the human genome, the majority of which were located within genes and their flanking regions, G-rich regions, and disease-associated genomic loci. The application of computational biology has provided novel perspectives and methodologies for R-loop research, contributing to a deeper understanding of their in vivo functions and mechanisms. These computational prediction methods not only allow for the identification of potential R-loop locations within the genome but also offer clues to R-loop function by analyzing the relationship between RLFS and other genomic elements. Studying RLFS helps elucidate the mechanisms by which R-loops participate in biological processes such as gene expression regulation and the maintenance of genome stability, providing a crucial theoretical foundation for further experimental investigation.

### 5.2. Development of network models for R-loop regulation

Constructing network models for R-loop regulation facilitates a systematic understanding of their regulatory mechanisms in vivo.^[59]^ For example, methods such as Lasso regression models, integrating information like sequence prediction data, miRNA co-regulation, RISC availability, and miRNA/mRNA abundance data, can identify miRNA-mRNA targeting relationships, thereby enabling the construction of miRNA regulatory networks. Analogous approaches can be employed in R-loop research to develop network models of R-loop regulation.

By studying patients with chronic obstructive pulmonary disease (COPD), researchers utilized machine learning algorithms to screen a large pool of R-loop regulators (RLRs), identifying 14 hub RLRs associated with COPD.^[60]^ Based on these identified RLRs, a risk score model was developed to stratify COPD patients into different subtypes. This work not only contributes to understanding the role of R-loops in the pathogenesis and progression of COPD but also provides an example for the construction of R-loop regulatory network models. Building such network models enables a more comprehensive understanding of the interactions between R-loops and other biomolecules, offering a powerful tool for in-depth investigation into the biological functions and disease associations of R-loops.

## 6. Discussion

Recent studies have shown that R-loops play an important role in inflammatory responses and immune escape of tumor cells. In addition, R-loops have been shown to play a regulatory role in antitumor immunity. Many genes can regulate the expression of immune checkpoints, and altering the expression of these genes may effectively improve the efficacy of immunotherapy.^[61]^ For example, JAK2 plays an important role in cell signaling, and its deletion is associated with a significant decrease in PD-L1 expression, which may improve the efficacy of PD-L1-targeted therapy.^[62]^ Inhibition of BRAF can be combined with PD-L1-targeted therapy to enhance the antitumor immune response. In addition, the combination of BRAF and PD-L1 inhibitors has shown promising synergistic therapeutic effects in some tumors.^[63]^ Therefore, combining R-loop-targeted therapy with existing immune checkpoint inhibitors, such as anti-PD-1 antibodies, may enhance the treatment effect. In a study on a mouse model of tumor, tumor growth rate, immune cell infiltration, and survival rate were analyzed to evaluate the effects of R-loop-targeted therapy.^[64]^ Compared with regulating the expression of a single gene, targeting R-loops can help regulate the expression of several genes simultaneously, which may produce a stronger therapeutic effect, improving the treatment response of patients.

Tumor metabolism and immunity are major hotspots in research related to tumor immune escape. In the TME, fierce metabolic competition exists between tumor cells and immune cells. To obtain nutrients required for growth, tumor cells deplete the nutrients required by immune cells. This competition not only weakens the function of immune cells but also promotes tumor cell growth and spread.^[65,66]^ For example, tumor cells inhibit T cell activity and proliferation^[67]^ by increasing the production of lactate, leading to acidification of the TME. Accumulation of R-loops is closely related to the metabolic reprogramming of tumor cells. Studies have shown that formation of R-loops can affect the energy production and nutrient utilization of cells by altering metabolic pathways. For example, R-loops may promote the upregulation of metabolic pathways such as glycolysis to provide tumor cells with the required energy and biosynthetic precursors.^[12]^ On the contrary, enhanced glycolysis leads to excessive lactate production, which eventually inhibits T cell activity in the TME. In addition, R-loops can interfere with mitochondrial function, affecting the oxygen utilization and energy generation^[10]^ of cells. We believe that R-loops can regulate more metabolic pathways and represent a novel avenue for further research on the TME.

Although the current relevant literature does not explicitly detail specific computational methods for identifying R-loop regulators, computational approaches hold potential value in this area, considering the overall progress in R-loop research.^[59]^ As investigation into R-loop regulatory mechanisms deepens, the accumulation of substantial experimental data provides a foundation for computational analysis. By integrating multi-omics data, including genomics, transcriptomics, and proteomics data, and utilizing machine learning and data mining algorithms, it may be possible to develop computational models capable of predicting R-loop regulators. For example, 1 can analyze gene expression patterns associated with R-loop formation and resolution to identify key regulatory factors therein. Computational methods can then be used to predict the regulatory effects of these factors on R-loops under different conditions. Furthermore, computational simulation of the structure and function of R-loop binding proteins can also help identify potential R-loop regulators, providing direction for further experimental validation and thereby contributing to a deeper elucidation of the R-loop regulatory network.

Developing R-loop-based immunotherapies and molecularly targeted therapies is of great clinical significance. However, genes located upstream of existing R-loop-targeted strategies remain unknown. Inhibiting these genes is necessary to restore normal gene expression and immune response. However, this method has uncertain inhibition efficiency and may require a long time to translate into clinical practice. Batch screening of small-molecule libraries is an important step in the field of drug discovery and chemical biology, which aims to quickly identify compounds with potential biological activities.^[68]^ Small-molecule libraries are usually composed of a series of structurally diverse compounds that are used to screen and identify potential drug candidates during drug development. The selection of and access to these libraries are essential for drug discovery because they provide a wide range of chemical classes for the exploration of therapeutic targets.^[69]^ We propose that a small-molecule library should be used to screen small-molecule inhibitors of R-loops. Virtual screening refers to a method based on drug design theory that utilizes computer technology and specialized application software to select promising compounds from a large number of compounds for experimental activity evaluation.^[70]^ Its purpose is to identify new lead compounds from tens to potentially millions of molecules. This drug screening method does not consume samples, thereby reducing screening costs. It also considers the pharmacokinetic properties and toxicity of the compound molecules, enhancing the depth of the screening process. We can first use virtual screening to obtain compounds that effectively inhibit R-loops, followed by further screening through the synthesis of customized compounds. This approach may not only help improve the efficiency of R-loop inhibition but also accelerate the clinical translation of R-loop-targeted drugs.

## Author contributions

**Writing – original draft:** Fan Zhang, Dingyun Wang, Guodong Zhao.

**Writing – review & editing:** Dingmao Wang.
